# Signatures of inflammation and impending multiple organ dysfunction in the hyperacute phase of trauma: A prospective cohort study

**DOI:** 10.1371/journal.pmed.1002352

**Published:** 2017-07-17

**Authors:** Claudia P. Cabrera, Joanna Manson, Joanna M. Shepherd, Hew D. Torrance, David Watson, M. Paula Longhi, Mimoza Hoti, Minal B. Patel, Michael O’Dwyer, Sussan Nourshargh, Daniel J. Pennington, Michael R. Barnes, Karim Brohi

**Affiliations:** 1 Centre for Translational Bioinformatics, William Harvey Research Institute, Barts and The London School of Medicine and Dentistry, Queen Mary University of London, London, United Kingdom; 2 Centre for Trauma Sciences, The Blizard Institute, Barts and The London School of Medicine and Dentistry, Queen Mary University of London, London, United Kingdom; 3 Centre for Translational Medicine and Therapeutics, William Harvey Research Institute, Barts and the London School of Medicine and Dentistry, Queen Mary University of London, London, United Kingdom; 4 Heart Centre, William Harvey Research Institute, Barts and The London School of Medicine and Dentistry, Queen Mary University of London, London, United Kingdom; 5 Department of Genetics, Evolution & Environment, University College London, London, United Kingdom; 6 Centre for Microvascular Research, William Harvey Research Institute, Barts and The London School of Medicine and Dentistry, Queen Mary University of London, London, United Kingdom; 7 Centre for Immunobiology, Blizard Institute, Barts and The London School of Medicine and Dentistry, London, United Kingdom; Oregon Health and Science University, UNITED STATES

## Abstract

**Background:**

Severe trauma induces a widespread response of the immune system. This “genomic storm” can lead to poor outcomes, including Multiple Organ Dysfunction Syndrome (MODS). MODS carries a high mortality and morbidity rate and adversely affects long-term health outcomes. Contemporary management of MODS is entirely supportive, and no specific therapeutics have been shown to be effective in reducing incidence or severity. The pathogenesis of MODS remains unclear, and several models are proposed, such as excessive inflammation, a second-hit insult, or an imbalance between pro- and anti-inflammatory pathways. We postulated that the hyperacute window after trauma may hold the key to understanding how the genomic storm is initiated and may lead to a new understanding of the pathogenesis of MODS.

**Methods and findings:**

We performed whole blood transcriptome and flow cytometry analyses on a total of 70 critically injured patients (Injury Severity Score [ISS] ≥ 25) at The Royal London Hospital in the hyperacute time period within 2 hours of injury. We compared transcriptome findings in 36 critically injured patients with those of 6 patients with minor injuries (ISS ≤ 4). We then performed flow cytometry analyses in 34 critically injured patients and compared findings with those of 9 healthy volunteers. Immediately after injury, only 1,239 gene transcripts (4%) were differentially expressed in critically injured patients. By 24 hours after injury, 6,294 transcripts (21%) were differentially expressed compared to the hyperacute window. Only 202 (16%) genes differentially expressed in the hyperacute window were still expressed in the same direction at 24 hours postinjury. Pathway analysis showed principally up-regulation of pattern recognition and innate inflammatory pathways, with down-regulation of adaptive responses. Immune deconvolution, flow cytometry, and modular analysis suggested a central role for neutrophils and Natural Killer (NK) cells, with underexpression of T- and B cell responses.

In the transcriptome cohort, 20 critically injured patients later developed MODS. Compared with the 16 patients who did not develop MODS (NoMODS), maximal differential expression was seen within the hyperacute window. In MODS versus NoMODS, 363 genes were differentially expressed on admission, compared to only 33 at 24 hours postinjury. MODS transcripts differentially expressed in the hyperacute window showed enrichment among diseases and biological functions associated with cell survival and organismal death rather than inflammatory pathways. There was differential up-regulation of NK cell signalling pathways and markers in patients who would later develop MODS, with down-regulation of neutrophil deconvolution markers. This study is limited by its sample size, precluding more detailed analyses of drivers of the hyperacute response and different MODS phenotypes, and requires validation in other critically injured cohorts.

**Conclusions:**

In this study, we showed how the hyperacute postinjury time window contained a focused, specific signature of the response to critical injury that led to widespread genomic activation. A transcriptomic signature for later development of MODS was present in this hyperacute window; it showed a strong signal for cell death and survival pathways and implicated NK cells and neutrophil populations in this differential response.

## Abstract 2

In a prospective cohort study, Joanna Shepherd and colleagues use whole blood transcriptome and flow cytometry analyses to identify cell populations and genes associated with a focused immune response very early after injury that develops into a widespread immune dysregulation and multiple organ dysfunction.

## Author summary

**Why was this study done?:**

- Multiple Organ Dysfunction Syndrome (MODS) is common in patients who survive critical injuries and is associated with poor patient outcomes, including death, infection, and prolonged critical care admission.
- MODS describes a failure of multiple organ systems (including lung, heart, kidney, and liver), and an excessive or dysfunctional immune response has been implicated in its development after trauma.
- The precise immune mechanisms leading to MODS are not fully understood, but the first minutes to hours after severe injury are likely to be pivotal to the development of a “normal” or “dysregulated” immune response.
- Our study was designed to investigate the very early immune responses to critical injury to determine whether a specific immune reaction occurs in the hyperacute timeframe that leads to widespread dysregulation and MODS.

**What did the researchers do and find?:**

- We studied 29,385 immune cell genes within whole blood samples obtained from 36 critically injured patients at admission (within 2 hours of injury) and compared these to samples obtained at 24 and 72 hours following injury.
- We analysed the differences between critically injured patient who developed MODS and those who did not and compared these to 6 patients who had minor injuries.
- We also used flow cytometry to analyse the numbers of circulating immune cell populations in 34 critically injured patients and compared these to healthy volunteers.
- Our study identified only 1,239 (4%) immune cell genes that were different between critical and control patients at admission; however, this subsequently developed into a widespread reaction by 24 hours postinjury.
- Comparing patients with MODS to those without MODS, we found 363 genes were different at admission, but by 24 hours postinjury, only 33 genes differentiated between the groups.
- Further analysis of the hyperacute timeframe demonstrated enrichment of gene pathways associated with cell death in patients with MODS and implicated neutrophils and natural killer immune cells in this response.

**What do these findings mean?:**

- The hyperacute timeframe is crucial to understanding the immune response to trauma and how this subsequently develops into MODS.
- There is a focused immune response to trauma in the hyperacute timeframe, which subsequently develops into a widespread immune reaction.
- The MODS signal was strongest in the hyperacute window and implicated cell death pathways and innate immune cells in this response.

## Introduction

The human immune response to injury is complex and vital to survival. An excessive, inappropriate, or dysfunctional immune response leads to cellular injury and Multiple Organ Dysfunction Syndrome (MODS), including lung, kidney, liver, and cardiovascular failure [1]. MODS contributes to the deaths and morbidity of most critically injured patients who survive the initial physical insult [2–3]. Patients with MODS require intensive care support, and this contributes significantly to the health outcomes and costs of injury [3]. As trauma is one of the leading contributors to the global burden of disease [4–6], understanding the immune responses to critical injury, and how dysregulation leads to adverse outcomes, is a major imperative in modern medicine.

The first minutes or hours after injury are likely to be pivotal to the development of a “normal” or dysregulated immune response and may set the trajectory for healthy or adverse outcomes. However, this hyperacute phase is very challenging to study in human patients due to the complexity and logistics of the emergency environment. Existing studies, therefore, tend to focus on patients later in their clinical course, after widespread inflammation has already been established and after patients have been subjected to further surgery, drugs, and transfusions containing other blood cell material [7]. This has potentially contributed to the ongoing controversies around the pathophysiology of organ dysfunction after trauma. Various models have been suggested, including a bimodal pro-inflammatory/anti-inflammatory profile [8]; a second-hit model of priming followed by exacerbation [9]; and, most recently, a widespread simultaneous activation of most of the circulating white cell transcriptome, termed a “genomic storm” [10]. The end-result of all of these models is a response involving so much of the immune system that its cumulative effect is unknown, and it is impossible to identify therapeutic targets for modulation of the inflammatory response. It is likely, however, that this widespread activation starts as a more focused and potentially unique observed response to trauma. Studies of the hyperacute response of the coagulation system have completely altered our understanding of the haemostatic response to injury [11], leading to new resuscitation paradigms and dramatic improvements in outcomes [12–14]. Overcoming the logistical challenges for robust inflammation research in the hyperacute phase may have a similar impact on our understanding of the human inflammatory response. It may be possible to identify the early mechanisms that then evolve into the full genomic storm—specifically, the early processes that lead to the development of MODS.

The overall objective of this study was to investigate the response of the whole blood transcriptome in critically injured patients in the hyperacute (within 2 hours) time window. The first aim of this study was to investigate the initial response to severe injury and whether there is an early, focused reaction. We aimed to determine the genes and pathways activated in leukocytes immediately after injury and then to describe how this evolves to become a widespread transcriptional activation, with a particular focus on the potential activation triggers. We further aimed to identify whether patients who developed MODS had a specific signature of transcriptome activation in the hyperacute phase, or whether the development of MODS simply reflects the magnitude of the physical insult and does not involve a differential response in the hyperacute window.

## Materials and methods

### Study setting and participants

The National Health Service (NHS) Research Ethics Committee (REC) granted ethical approvals for this research (07/Q0603/29, 13/LO/0363). The research was a substudy of our prospective platform cohort study, Activation of Coagulation and Inflammation in Trauma (ACIT2). All samples and clinical data were collected prospectively. The analysis plan for this substudy was developed after patient enrolment and sample acquisition (S1 Text). The study is reported according to STROBE Guidelines (S1 Table). ACIT2 is a prospective study evaluating aspects of coagulation and inflammation in trauma patients (NHS REC: 07/Q0603/29). All adult trauma patients (older than 15 years of age) at The Royal London Hospital who met the local criteria for trauma team activation were eligible for enrolment when research personnel were present (during this period 08:00 to 22:00 daily). Criteria for trauma team activation are based on a high-energy mechanism of injury (including fall greater than 3 m, road traffic collision greater than 30 mph, pedestrian/cyclist/motorcyclist versus vehicle, ejection from vehicle, fatality in same vehicle as occupant, entrapment, gunshot wound, major crush injury, or blast injury), abnormal patient physiology (including intubated patient, Glasgow Coma Score less than 14, respiratory rate less than 10 or greater than 30, systolic blood pressure less than 90, or heart rate greater than 100), or high-risk anatomical injury patterns. Inclusion and exclusion criteria for ACIT2 are described elsewhere [15]. All patients or their representatives provided written informed consent for study inclusion.

### Sample and data collection

Data collected on each patient included measures of shock and tissue ischemia (base deficit and lactate) and injury severity as assessed by the Injury Severity Score (ISS) [16] and the Abbreviated Injury Scale (AIS) [17]. Admission bloods were drawn immediately on arrival in the resuscitation room and within 2 hours of injury. For genomic analysis, whole blood was collected in 2.5 ml PAXGene Blood RNA tubes (Pre-AnalytiX GmBH, Switzerland) and stored as recommended by the manufacturer. For lymphocyte subpopulation phenotyping (B, T, and Natural Killer [NK] cells), whole blood was collected in either 8.0 ml CPT, Vacutainer or 6.0 ml EDTA Vacutainer tubes (Becton Dickinson, UK). Temporal bloods were drawn at 24 and 72 hours following admission. Patient outcomes, including 28-day mortality, infection, critical care, and hospital length of stay, were prospectively recorded.

### Patient selection

Of the enrolled ACIT2 patients, critically injured patients were selected for inclusion into microarray and flow cytometry studies if they sustained severe trauma (ISS ≥ 25) with a blunt mechanism of injury. A cohort of trauma patients suffering minor injuries (ISS ≤ 4) served as a control cohort. Patients were excluded from microarray studies if they received any blood products or greater than 1000 ml of prehospital crystalloid prior to their admission blood draw, or if they sustained severe traumatic brain injuries (defined as a head AIS score > 3). These predefined selection criteria were established to counter the influence of blood products and iatrogenic intervention on the immune response and the influence of severe traumatic brain injury on outcome. The principle outcome measure was MODS, defined as a sequential organ failure assessment (SOFA) score of ≥ 5 on 2 or more consecutive days, excluding the first 48 hours [18–19].

Patients for the transcriptomic analysis were enrolled to ACIT2 between December 2008 and June 2012. Overall, 556 patients were enrolled to ACIT2 of which 90 had blunt trauma, ISS ≥ 25, and no traumatic brain injury. Of these, 36 patients had samples at all 3 time points and formed the critical population for this study. The critical population was dichotomised based on the presence (MODS: 16 patients) or absence (NoMODS: 20 patients) of MODS. Patients for the flow cytometry analysis were prospectively enrolled into ACIT2 between June 2014 and January 2017. A total of 404 patients were recruited to ACIT2 in this time period of which 175 were critically injured and met criteria for this study. For 34 of these patients, a flow cytometry trained researcher was present and there was timely access to the flow cytometer. A cohort of 9 healthy volunteers (NHS REC: 13/LO/0363) served as a control population for the flow analyses. These sample sizes represented convenience samples, as there was no prior data available to power the studies.

### Flow cytometry

Peripheral blood mononuclear cells (PBMCs) were isolated from CPT^™^ tubes (Beckton Dickinson, UK) using the manufacturer’s instructions. EDTA anticoagulated blood was treated with Pharm Lyse (Beckton Dickinson) for the removal of erythrocytes. Freshly prepared mononuclear cells or lysed cells were washed in phosphate buffered saline (Thermofisher Scientific, UK) twice and resuspended in staining buffer (Dulbecco’s phosphate buffered saline, with 2% Fetal Bovine Serum, [Stemcell, France]) prior to staining. Lymphocyte subpopulations were identified using combinations of the following antibodies: CD3-V450 (UCHT1), CD56-CF-594 (NCAM 16.2), CD16-BV510 (3G8), CD14-APC-Cy7 (MoP3), CD19-APC-Cy7 (SJ25CI), CD4-PerCP-Cy5.5 (SK3), CD8-APC-H7 (SK1), and CD19-PerCP-Cy5.5 (HIB19) (Beckton Dickinson, UK). After staining, samples were washed in PBS and fixed in 2% paraformaldehyde solution. Acquisition was performed using a LSR II flow cytometer (Beckton Dickinson, UK).

### Data analysis

#### Microarray protocols

Total RNA was extracted from whole blood collected in PAXGene Blood RNA tubes (Pre-AnalytiX GmBH, Switzerland) as previously described [20]. Quantity and integrity was assessed [20]. Total RNA (250 ng) was amplified using the Ambion TotalPrep Kit (Illumina, USA), and 750 ng of the resulting cRNA was hybridised to the Illumina Human HT-12 v4 Expression Beadchip (Illumina, USA). Arrays were scanned using the Illumina iScan 2.0 system. Data were exported to R for statistical analysis using Illumina Genome Studio V2011.1. ComBat [21] from the surrogate variable analysis (SVA) package in Bioconductor was used to remove known batch effects. Background correction and quantile normalisation using control probes was applied to the data using the neqc function of the linear models for microarray data package (limma) [22]. Intensities were log2 transformed and control probes removed. Nonexpressed probes were filtered out, keeping only probes with a detection p-value of 5% in 3 arrays or more. The prcomp function from R was used to perform principal component analysis (PCA) on gene expression values to assess patient stratification. In addition, gene expression patterns were further evaluated using Euclidean distance heatmaps. Differentially expressed genes between patient categories (e.g., critical versus controls at 0 hours) were identified using linear models and empirical Bayes methods [23]. Intraclass correlations were estimated to account for repeated measures on the same patients [24]. p-values were adjusted for multiple testing using the Benjamini-Hochberg false discovery rate (FDR) [25]. Genes with an FDR ≤ 0.05 were considered differentially expressed and utilised for further analysis. Raw and normalized gene expression data is available from ArrayExpress under E-MTAB-5882. All data files and analysis code are also available from the following repository: https://github.com/C4TS/HyperacutePhase.

#### Pathways and networks

Ingenuity pathway analysis software (IPA) (Qiagen, inc) was used to identify biological mechanisms enriched in the differentially expressed genes. This software analyses transcriptomic data in the context of known pathways and regulatory networks, identifying biological functions and/or pathways that are significantly enriched in the results. Genes from the dataset that met a *p* < 0.05 cut-off and were associated with biological functions in the Ingenuity Pathways Knowledge Base were analysed. Significance of the biofunctions and the canonical pathways were tested by a Fisher Exact test p-value, to exclude the probability of enrichment by chance alone. Pathways were grouped by the ratio value (the number of significant molecules in each pathway divided by the total number of molecules that make up that pathway). Gene coexpression networks were built for the most enriched pathways to assess the progression of changes in differentially expressed genes and their clustering patterns. These coexpression networks were created using BioLayout Express3D [26], applying a minimum threshold of 0.7 Pearson correlation score.

#### Immune cell deconvolution

To estimate the immune cell composition of our samples, we downloaded the immune response in silico (IRIS) repository of 1,622 genes specifically expressed in, and classified by, multiple immune cell lineages [27]. Differentially expressed, lineage-specific transcripts were used to estimate changes in cell composition in samples, based on the percentage of lineage-specific transcripts present in each group at each time point. Cluster 3.0 was used to generate heatmaps of differentially expressed immune cell markers [28].

#### Immune module analysis

We examined the functional properties of transcriptomic signatures by focusing on coexpressed gene sets, previously modularised by Chaussabel et al. [29]. These 260 modules have been the subject of extensive study across a variety of inflammatory disorders, and biological annotations have been provided for many of them (module transcript content and annotations are available online at http://www.biir.net/public_wikis/module_annotation/V2_Trial_8_Modules) [30]. We tested for differential expression across modules using the QuSAGE [31–32]. The design for this portion of the study was identical to that for our limma analysis, with intraclass correlations for repeated observations estimated using a compound symmetry correlation structure [33].

#### Flow cytometry analysis

Patient demographic data were analysed in Excel (Microsoft, USA) and Prism (GraphPad, USA). Flow cytometric results were analysed using FlowJo (Tree Star, USA). Absolute counts for T cells, B cells, and NK cells were obtained by multiplying their percentage (of the total lymphocyte population acquired by flow cytometry) by the absolute lymphocyte count obtained from routine hospital laboratory tests. Data are principally reported as median (interquartile range) and analysed with nonparametric statistics unless otherwise stated.

## Results

The demographics and injury characteristics of the patient groups are shown in Table 1 and S1 Fig. Critically injured patients in the microarray cohort were severely injured with a median ISS of 33, compared to the control cohort with a median ISS of 1. No patient in this cohort received a blood transfusion or any surgical intervention prior to sampling. The initial blood sample was taken immediately on arrival in the emergency department, within 30 minutes and a median of 93 minutes after injury (within 2 hours of injury in all patients).

**Table 1:** Patient demographics.

		Microarray Cohort					Flow Cytometry Cohort	
Demographics	Control^a^	Critical^b^	p-value^1^	NoMODS	MODS	p-value^2^	HV	Critical^b^
Number	6	36	–	16	20	–	9	34
Age†	40 (29–46)	36 (26–53)	0.90	33 (23–41)	39 (26–63)	0.05	34 (30–42)	51 (33.5–61)
Male (%)††	83	75	1.00	88	62	<0.01	60	71
Time from injury to blood draw (mins)†	59 (36–83)	93 (72–111)	<0.01	80 (68–97)	102 (85–115)	0.04	–	105 (90–117)
**Injury characteristics**								
ISS†	1 (0–2)	33 (27–39)	<0.01	29 (27–33)	38 (29–43)	0.06	–	38 (29–45)
SBP on admission†	134 (121–152)	98 (82–126)	0.06	111 (96–138)	94 (66–124)	0.05	–	112 (73–132)
Preintubation GCS†	15 (14–15)	14 (13–15)	0.67	15 (14–15)	14 (13–15)	0.33	–	8 (3–14)
BD (mmol/L)†	−1.1 (-1.7–−0.6)	5.8 (2.7–9.8)	0.01	2.5 (2.0–3.8)	9.7 (6.3–15.3)	<0.01	–	7.3 (4.3–16.9)
Shock Index (HR/SBP)†	0.7 (0.5–0.7)	1.3 (0.7–1.7)	0.20	0.8 (0.7–1.1)	1.7 (1.4–2.3)	0.02	–	0.9 (0.7–1.1)
Lactate (mmol/L)†	2.2 (1.6–2.3)	4.4 (2.2–7.2)	0.08	2.0 (1.4–4.1)	6.6 (4.4–11.5)	<0.01	–	3.2 (2.1–9.9)
AIS Head and Neck†	0 (0–0)	0 (0–1)	0.31	0 (0–0)	0 (0–2)	0.08	–	2 (0–4)
AIS Face†	0 (0–1)	0 (0–0)	0.25	0 (0–0)	0 (0–0)	0.30	–	0 (0–1)
AIS Thorax†	0 (0–0)	4 (3–5)	<0.01	4 (3–5)	4 (4–5)	0.31	–	3 (3–4)
AIS Abdo/Pelvis†	0 (0–0)	2 (0–4)	<0.01	3 (2–4)	2 (0–3)	0.05	–	3 (2–4)
AIS Extremity/Pelvis†	0 (0–0)	3 (2–3)	<0.01	3 (2–3)	3 (3–3)	0.03	–	3 (2–4)
**Outcomes**								
28-day mortality (%)††	0	19	0.57	0	33	<0.01	–	29
Infections (%)††	0	73	<0.01	44	86	<0.01	–	48
Hospital stay (days)†	4 (3–6)	18 (12–34)	0.11	16 (12–21)	24 (12–44)	0.23	–	13 (2–45)
ICU stay (days)†	0	6 (1–15)	0.02	1 (0–4)	15 (9–18)	<0.01	–	6 (2–21)

Median (interquartile range) reported unless otherwise specified.

p-value comparisons in the microarray cohort include ^1^Control versus Critical and ^2^MODS versus NoMODS using ^†^Mann-Whitney U test and ^††^Fisher’s exact test.

^a^Minor injured trauma patient (ISS 0–4),

^b^critically injured trauma patient (ISS ≥ 25).

AIS, Abbreviated Injury Scale; BD, base deficit; GCS, Glasgow Coma Score; HR, heart rate; HV, healthy volunteer; ICU, intensive care unit; ISS, Injury Severity Score; MODS, Multiple Organ Dysfunction Syndrome; NoMODS, did not develop MODS; SBP, systolic blood pressure

### The hyperacute immune response to injury

**Fig 1:**
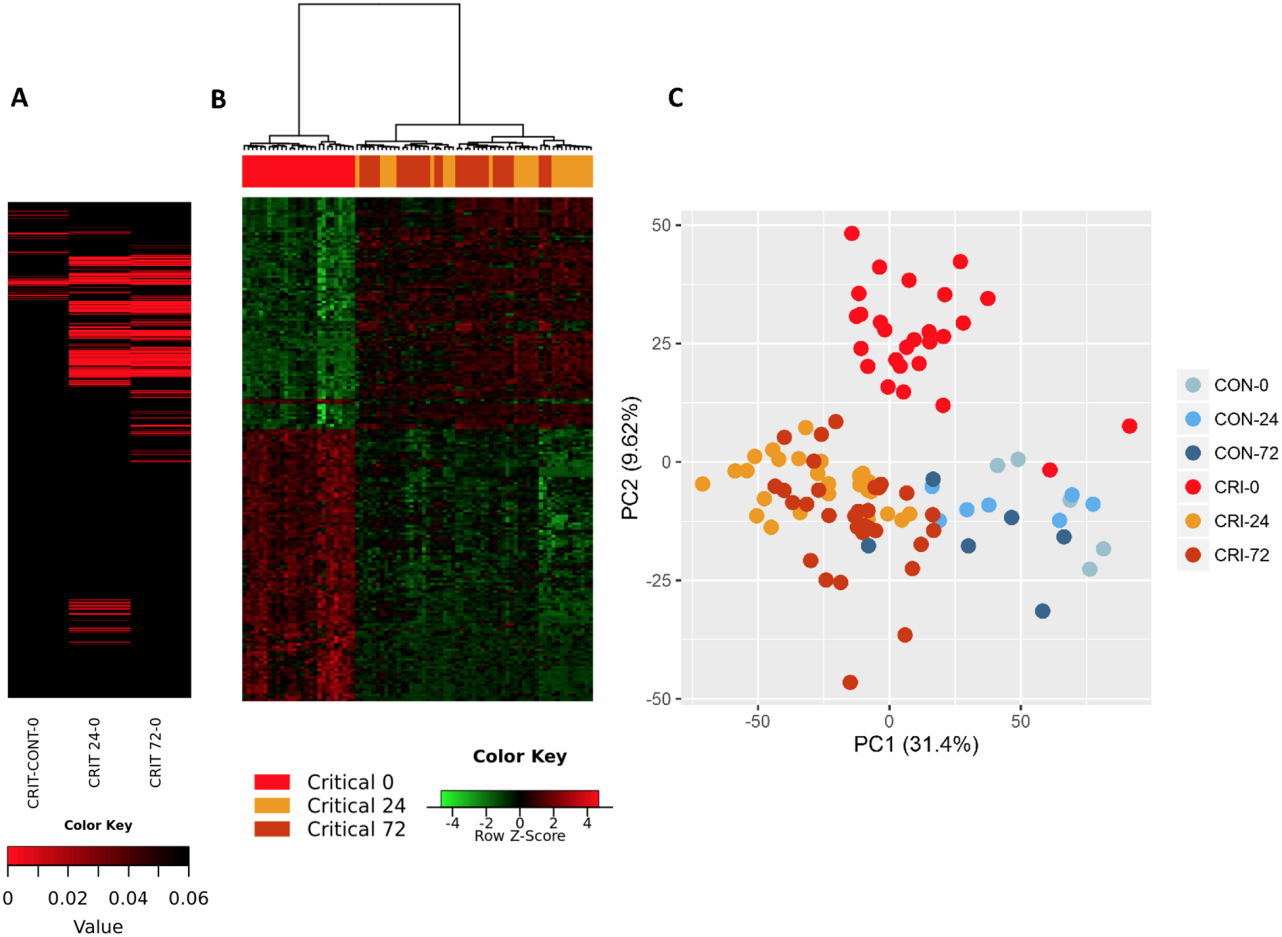
The hyperacute transcriptomic response to critical injury. (A) There is a focused genomic response to injury in the hyperacute window after trauma. Columns show log p-values of differentially expressed genes between 36 Critical and 6 Control patients at 0 hours postinjury (hyperacute window), and between Critical patients at 24 and 0 hours and at 72 and 0 hours. In the hyperacute window, 4% of transcripts (1,239 of 29,385) were differentially expressed. This expanded to 21.4% (6,294 transcripts) at 24 hours and 21% (6,177 transcripts) at 72 hours. (B) Cluster analysis of differentially expressed genes in critically injured patients (versus controls). All hyperacute samples clustered separately, whereas there was no differentiation between samples at 24 or 72 hours. (C) Principal component analysis of differentially expressed genes in Critical and Control patients at 0 (hyperacute window), 24, and 72 hours postinjury. Critical patients were well separated from control patients, and there was marked separation of the hyperacute samples.

Within the immediate 2-hour hyperacute time window, we observed a focused signature of leukocyte gene expression across critically injured patients. Only 1,239 (4.2%) of the total 29,385 gene probes were differentially expressed at this time point compared to controls (FDR ≤ 5%). By 24 hours postinjury, this response had blossomed into a much more widespread transcriptional activation (Fig 1A). A total of 6,294 genes (21.4%) showed differential expression at 24 hours postinjury compared to admission, and this expression pattern persisted at 72 hours, at which time 6,177 genes (21.0%) remained differentially expressed compared to the admission sample (Fig 1A). In contrast, there was strong concordance between later expression profiles, with 4,094 (65%) of the 6,294 24-hour differentially expressed genes still differentially expressed at 72 hours (Fig 1B). In a principal component analysis, this immediate gene activation showed good separation from control patients at all time points and critical patients at later time points (Fig 1C). The normal human response to critical injury begins with the differential expression of a small subset of genes but evolves rapidly and is maximal at the 24-hour postinjury time point.

**Fig 2:**
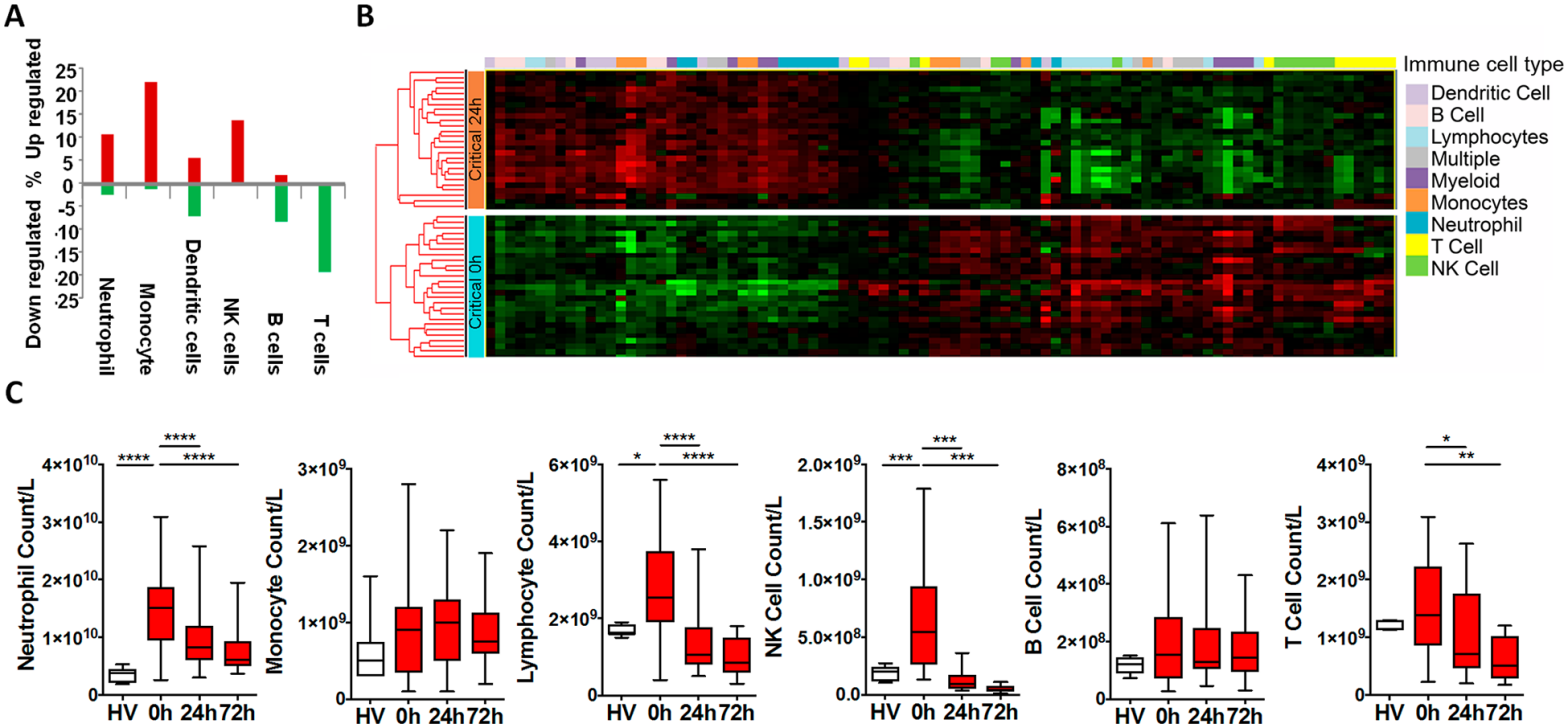
Leukocyte subpopulation changes in the hyperacute response to injury. (A) Immune cell deconvolution showing overall % differential regulation of immune cell—specific markers (selected from the immune response in silico [IRIS] resource [see methods]) between critical and control patients at 0 hours. There was predominant up-regulation of neutrophil, monocyte, and natural killer (NK) cell markers, a mixed-response in dendritic cells, and down-regulation of B and T cells. (B) Hierarchically clustered heatmap of immune cell—specific/enriched markers (selected from the IRIS resource) in the 36 critical patients at 0 and 24 hours postinjury. (C) Flow cytometry analyses were consistent with deconvolution. There was increase in numbers of neutrophils and lymphocytes (principally NK cells) in the hyperacute window. By 24 hours, total lymphocytes and NK cells were below normal (as compared to healthy volunteers), and median T-cell counts had also fallen significantly below healthy control counts. (Total leukocyte counts: healthy volunteer: 5.8 (4.4–7.1) x 10^9^/L; 0 hours: 19.5 (13.0–23.1) x 10^9^/L; 24 hours: 10.4 (7.8–14.1) x 10^9^/L; 72 hours: 8.2 (6.6–11.7) x 10^9^/L. *p < 0.05, **p < 0.01, ***p < 0.001, ****p < 0.0001).

We used immune cell in silico deconvolution methodology and flow cytometry (S2 Fig) to examine differential responses in leukocyte populations within the hyperacute window. Deconvolution identified up-regulation in marker genes for neutrophils, monocytes, and NK lymphocytes, a mixed response in B lymphocytes and dendritic cells, and down-regulation of T lymphocytes (Fig 2A). By 24 hours postinjury, the leukocyte signatures had developed a profoundly different pattern of activation (Fig 2B). Cell proportions by in silico deconvolution were broadly consistent with direct count data from flow analysis (Fig 2C). In the hyperacute window, we observed a dramatic rise in the lymphocyte subset, which was almost entirely accounted for by a 150% increase in the number of NK cells in the circulation (Fig 2C). By 24 hours, these changes had disappeared, and NK cell counts were half-normal at 24 hours and approached zero at 72 hours. By contrast, T-lymphocyte levels were maintained in the hyperacute window but then fell by 40% at 24 hours. Neutrophil counts also peaked in the hyperacute window, to nearly 4 times the level in healthy controls. The count then gradually declined over the later time points to less than twice normal at 72 hours (Fig 2C). Hyperacute monocyte and B cell counts were not significantly elevated. However, 20% of up-regulated immune transcriptome markers were of monocyte origin, while B cells showed primarily down-regulation in this early window (Fig 2A & 2C). Collectively, data on immune cell populations indicate a rapid change in neutrophil and innate lymphocyte populations in the hyperacute phase of critical injury. This is associated with principally up-regulation of the transcriptome of innate leukocyte populations and down-regulation of adaptive lymphocyte populations.

The hyperacute postinjury circulating leukocyte expression profile represents the earliest genes and pathways involved in the initiation of the systemic inflammatory response. Of the 1,239 genes differentially expressed in the hyperacute window, 666 (54%) were up-regulated and 573 (46%) down-regulated. By 24 hours postinjury, transcriptomic activation had grown in magnitude and evolved in character. The proportion of up-regulated (3,382 of 6,294, 54%) and down-regulated genes (2,912, 46%) was similar to the hyperacute phase. However, only 202 (16%) of genes differentially expressed in the hyperacute window were still differentially expressed in the same direction at 24 hours postinjury (122 upregulated and 80 downregulated). By 24 hours, 254 (20%) genes that were initially up-regulated were down-regulated, and 32 initially down-regulated genes were upregulated.

**Fig 3:**
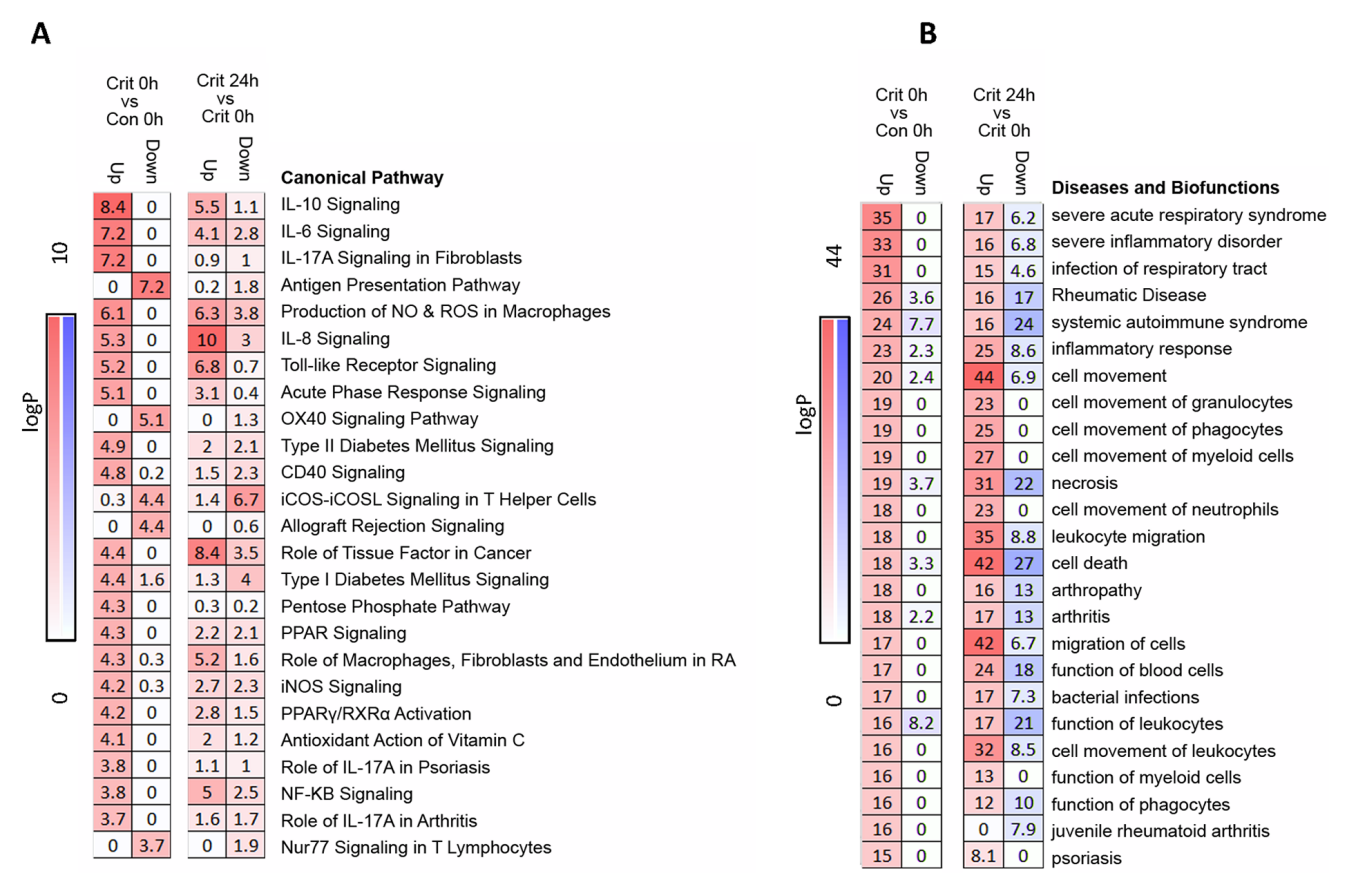
Pathways and biofunctions in the hyperacute response to critical injury. (A) Ingenuity pathway analysis of differentially expressed genes in the hyperacute window (Critical versus Control, 0 hours) proceeding to 24 hours postinjury (Critical 24 hours versus Critical 0 hours). Broadly, in the hyperacute window, there is up-regulation of innate inflammatory response and down-regulation of some adaptive immune pathways. By 24 hours, there is a much greater preponderance of down-regulated responses. (B) Ingenuity disease and biofunction analysis of differentially expressed genes in the hyperacute window (Critical versus Control, 0 hours) proceeding to 24 hours postinjury (Critical 24 hours versus Critical 0 hours). Again, there is immediate up-regulation of biofunctions related to inflammation and leukocyte movement, with down-regulation becoming more prominent at 24 hours.

**Fig 4:**
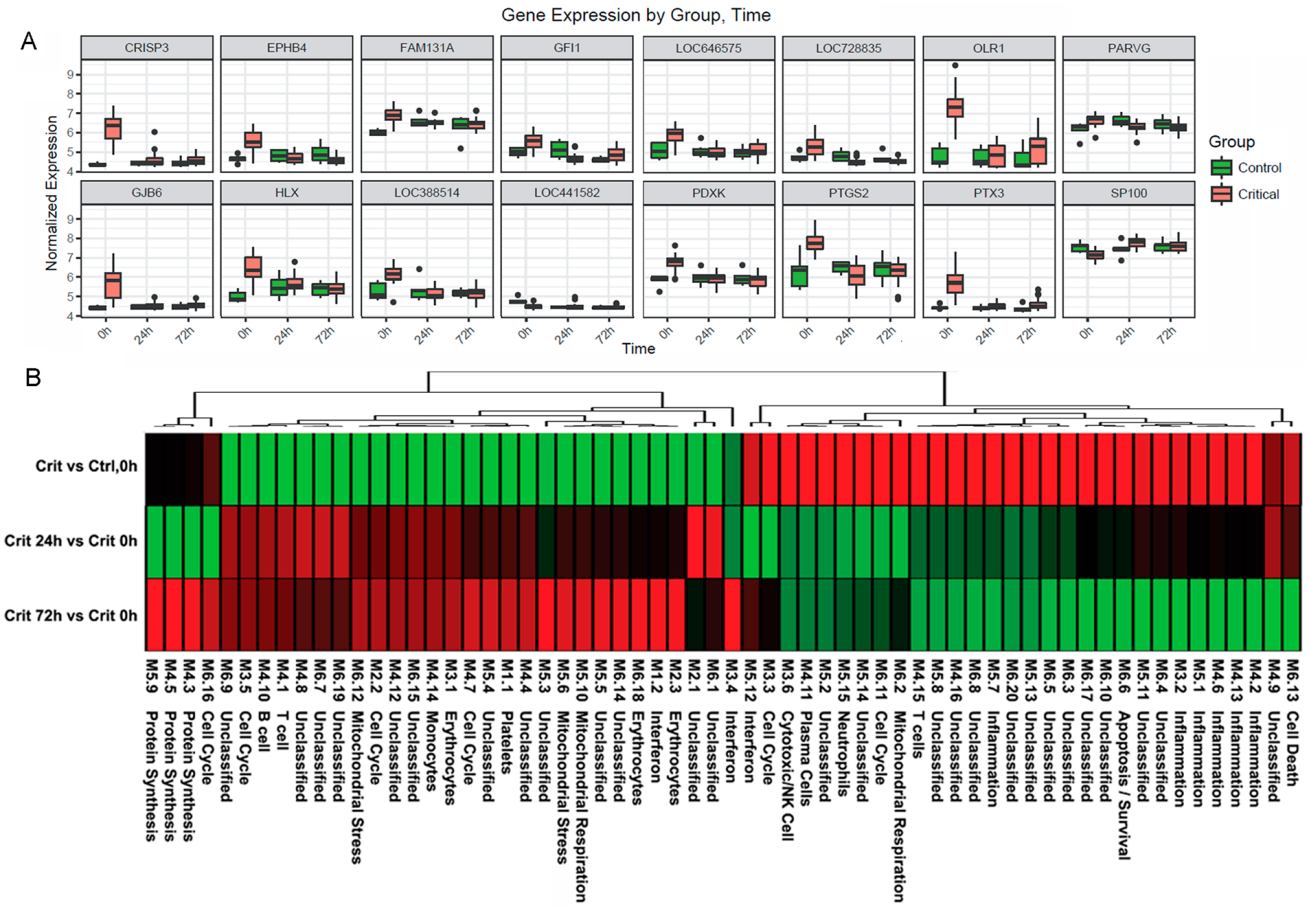
Immune systems analysis in the hyperacute window. (A) Gene expression profiles of the 16 genes with the greatest difference in expression between the hyperacute window and 24 hours postinjury in the Critical versus Control transcriptome analysis. (B) Immune module analysis of the critical response to injury. Again, there was a differential response between the hyperacute response and the later time points. There was overexpression in modules associated with inflammation and of neutrophil and natural killer (NK) cell modules. There were mixed over- and underexpression in modules related to T cells and B/plasma cells and underexpression of monocyte-related modules (contrary to the deconvolution data in Fig 2B).

Ingenuity canonical pathway analysis on genes differentially expressed in the hyperacute window demonstrated widespread up-regulation of innate inflammatory response pathways and biofunctions, including the IL-10, IL-6, IL-17A, and IL-8 signalling pathways; nitric oxide (NO) signalling; toll-like receptor signalling; and acute-phase response signalling pathways (Fig 3A & 3B, S2 Table). There was down-regulation of adaptive pathways such as antigen presentation, T-cell signalling (X40, iCOS, Nur77), and allograft rejection signalling. After 24 hours, there was a much greater preponderance of down-regulation across all pathways and biofunctions (Fig 3A & 3B). Fig 4A shows boxplots of exemplar genes from pathways that are differentially expressed in the hyperacute phase but which have changed their response by 24 hours.

To further characterise the biological processes that may be driving the hyperacute phase, we analysed the expression of 260 previously reported immune gene modules coexpressed in blood across a range of immunological conditions. Applying a 5% FDR threshold, we identified 118 modules differentially expressed between critical and control patients at baseline, 204 between critical patients at 24 hours postinjury versus 0 hours, and 187 between critical patients at 72 hours postinjury versus baseline (S2 Table). Functional interpretation by QuSAGE was used to generate a hierarchically clustered heatmap presenting log fold change of module expression across critical and control patients (Fig 4B). Of the 30 modules overexpressed in the hyperacute phase, 28 had normalised or become underexpressed by 24 hours postinjury, and 27 of 32 initially underexpressed modules were becoming overexpressed by 24 hours. No modules showed persistent over- or underexpression over the 72-hour period postinjury.

In the hyperacute phase, inflammation predominated with signature overexpression of modules M3,2. M4.2, M4.6, M4.13, M5.1, and M5.7, as well as modules annotated to neutrophils (M5.15) and cytotoxic/NK cells (M3.6). A mixed response was seen with modules annotated to T cells (M4.15) and B cells/plasma cells (M4.10, 4,11), while the monocyte module M4.14 was underexpressed. There was a mixed response across modules related to the cell cycle (M2.2, M3.3, M3.5, M4.7, M6.11, M6.16), mitochondrial functions (M5.6, M5.10, M6.2, M6.12), and interferon (M1.2, M3.4, M5.12). Detailed module annotation is linked in S2 Table. These results reinforce evidence for a specific hyperacute inflammatory response signature that changes to a very different pattern by 24 hours postinjury and may be driven by a differential response between innate (neutrophil and NK cell) and adaptive systems.

### Development of MODS

**Fig 5:**
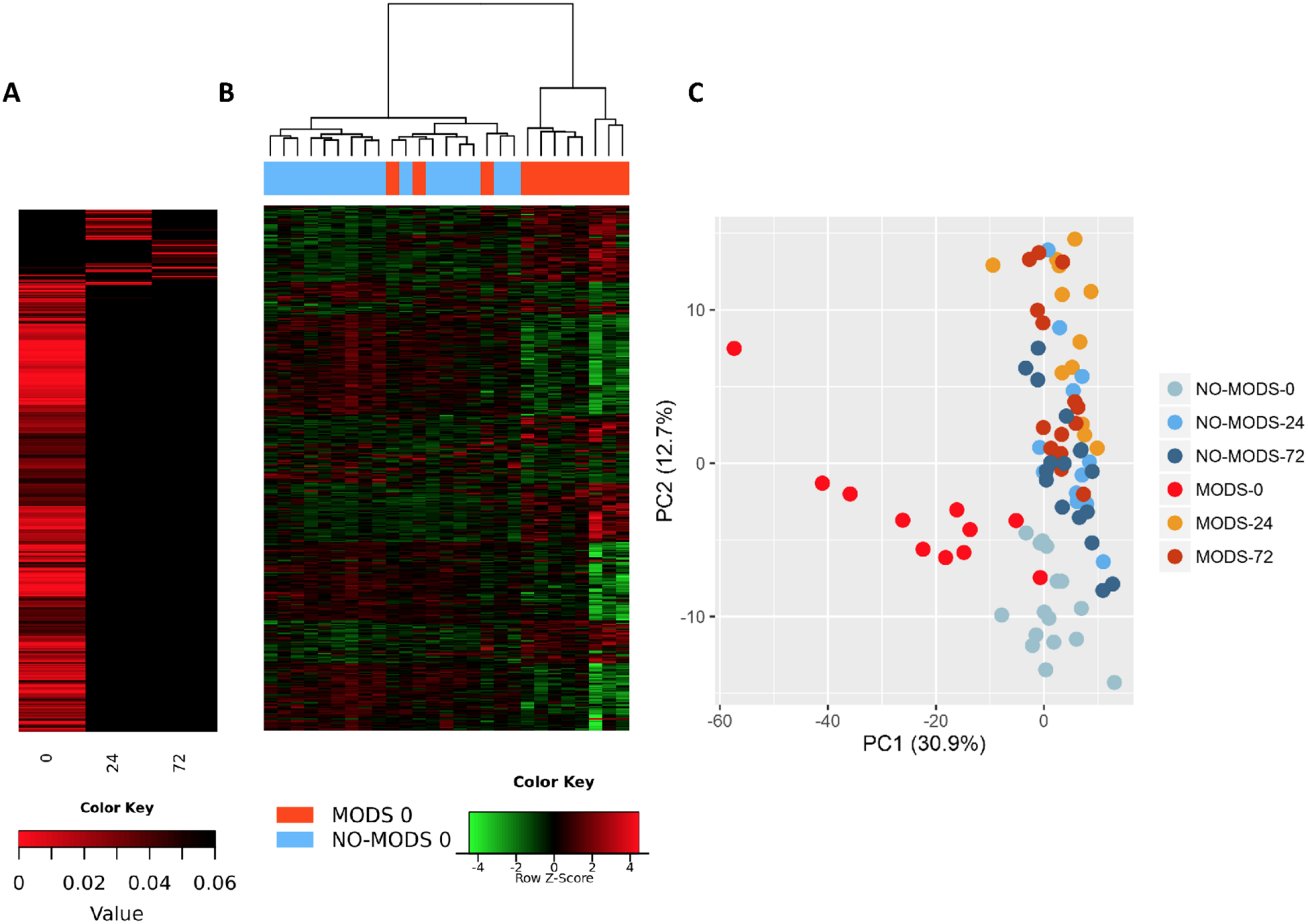
Patients who develop Multiple Organ Dysfunction Syndrome (MODS) have a specific differential gene expression only in the hyperacute window. (A) Differential expression heatmap with columns showing log p-values of all differentially expressed genes between 20 critical patients who later developed MODS versus 16 patients who did not develop MODS (NoMODS) at 0 hours (hyperacute time point), 24 hours, and 72 hours postinjury. At 0 hours, 363 transcripts were differentially expressed between MODS and NoMODS patients, and only 33 and 28 transcripts at 24 and 72 hours, respectively. (B) Cluster analysis of differentially expressed genes in MODS versus NoMODs patients in the hyperacute window. MODS patients clustered separately apart from 3 patients, 2 of which had a mild clinical phenotype that rapidly resolved. (C) Principal component analysis of differentially expressed genes in MODS versus NoMODS patients at 0 (hyperacute window), 24, and 72 hours postinjury. There was strong separation in the transcriptomic response to MODS in the hyperacute window.

**Fig 6:**
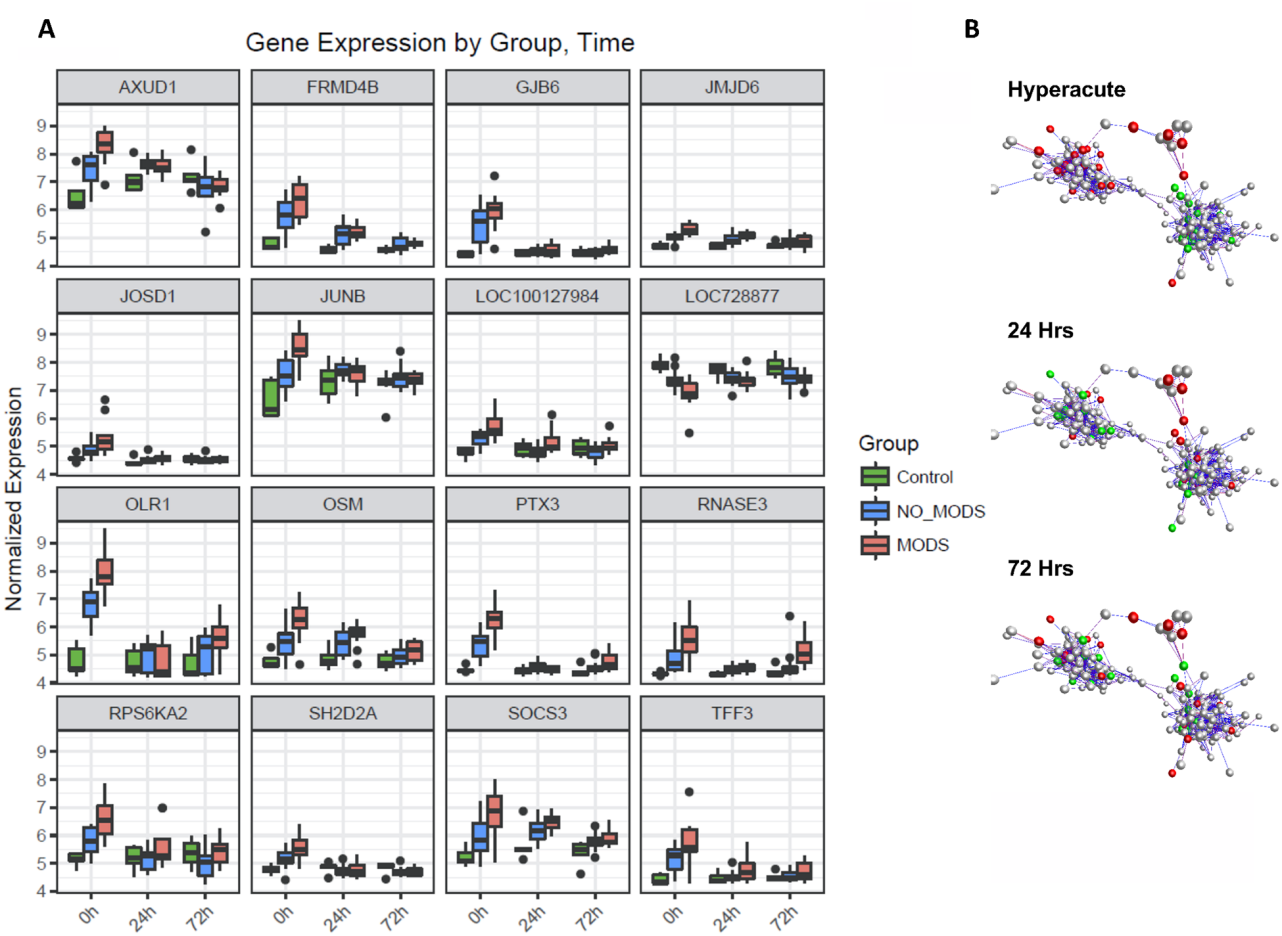
Differential gene expression of patients who later develop Multiple Organ Dysfunction Syndrome (MODS). (A) Gene expression profiles of the 16 genes with the greatest difference in expression between MODS and those that did not develop MODS (NoMODS), which were also differentially expressed in the Critical versus Control analysis. The greatest differences in MODS versus NoMODS expression are seen in the hyperacute (0 hours) time point in all cases. (B) Coexpression network representation of all differentially expressed MODS genes. Coloured genes are those mapped to cell death and organismal survival pathways. Red: up-regulated; green: down-regulated. Two tightly correlated clusters are represented, with cell death represented across both clusters. There is an almost complete reversal of expression pattern of these genes between the hyperacute window and 24 hours postinjury.

We wished to determine whether we could identify a transcriptomic signal for the development of MODS. We found that almost the entire differential response associated with later MODS development was present in the hyperacute window with very little change at 24 hours postinjury or thereafter (Fig 5). Within 2 hours of injury, 363 transcripts were differentially expressed in patients who subsequently developed MODS compared to those who did not. By 24 hours postinjury, only 33 genes were differentially expressed in MODS versus NoMODS patients, and 28 genes at 72 hours (Fig 5A). In cluster and principal component analyses, the hyperacute response in patients who later developed MODS was very different from both patients who never developed MODS and from MODS patients at later time points (Fig 5B & 5C). On review, 2 of the 3 MODS patients who clustered with the NoMODS patients had borderline SOFA scores, which had dropped below the threshold criteria for MODS by day 3 postinjury. The MODS signature is clearly set within the hyperacute window, and most of these genes either normalise or invert their levels of expression by 24 hours (Fig 6A & 6B).

**Fig 7:**
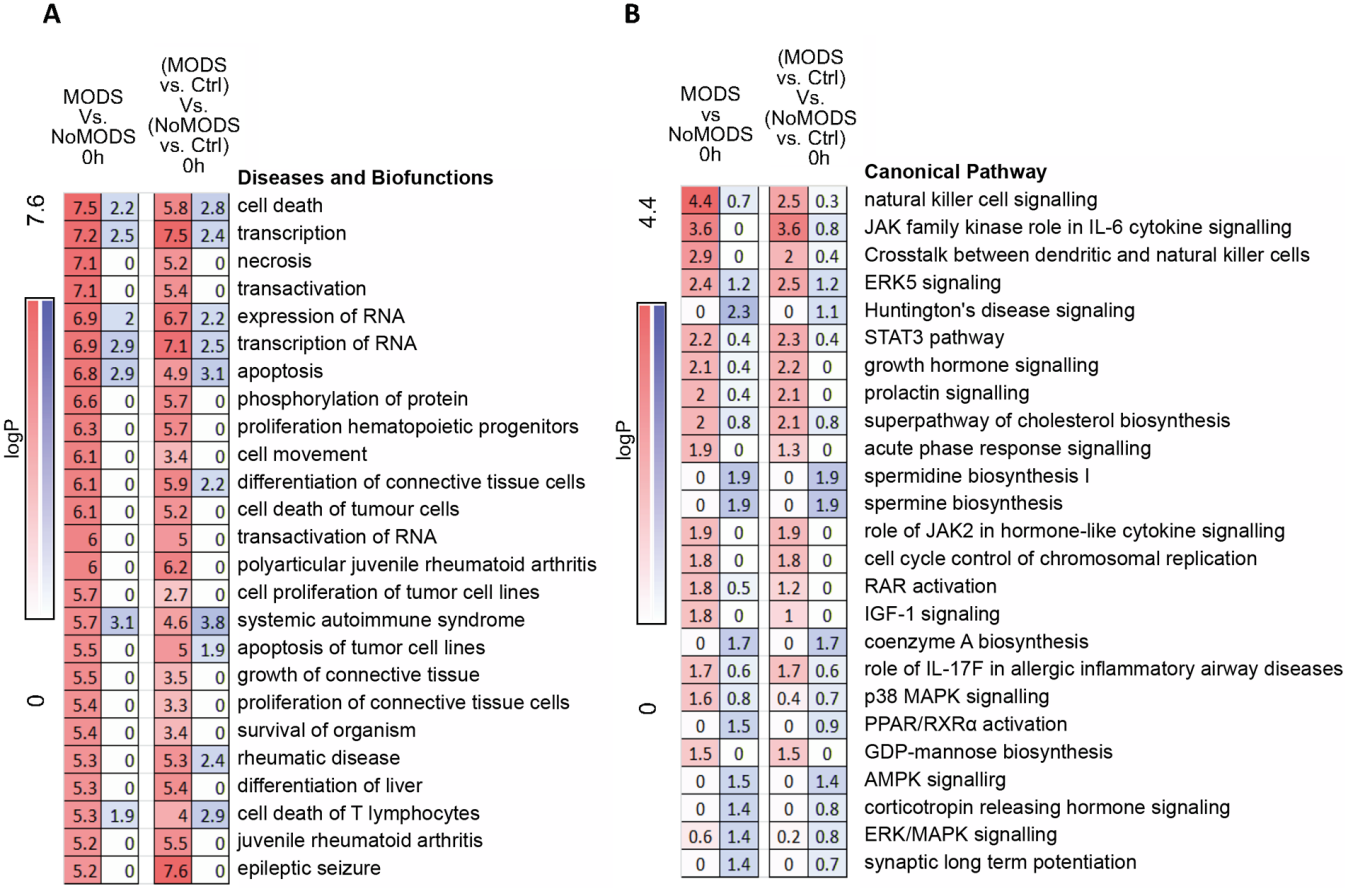
Pathways and biofunctions in the hyperacute response in patients who later develop Multiple Organ Dysfunction Syndrome (MODS) versus those who do not (NoMODS). (A) Ingenuity canonical pathway analysis of genes showing differential expression at 0 hours postinjury in patients who experienced MODS compared to those who did not (MODS versus NoMODS, 0 hours). (B) Ingenuity Diseases and Biofunctions analysis of genes showing differential expression at 0 hours in patients who experienced MODS compared to those who did not (MODS versus NoMODS, 0 hours). There is little overlap between the pathways identified in the MODS analysis and that of the response to critical injury analysis in Fig 3A. Only acute-phase response signalling appears in both analyses.

MODS transcripts differentially expressed in the hyperacute window showed enrichment among diseases and biological functions associated with cell survival and organismal death (Fig 7A). Differentially expressed genes were annotated to cell death, necrosis, or apoptosis functions in multiple cell lines. Only 20 (6%) of the transcripts were annotated to genes associated with an inflammatory response. In canonical pathway analysis, there was a distinct difference in the pathways activated in the critical response (Fig 3A), and those associated with the development of MODS (Fig 7B). While the classical genes and inflammatory pathways (e.g., IL-8, IL-10) did not appear in the analysis, many were related to the regulation or downstream signalling of these pathways (e.g., STAT3, ERK, and IGF-1 for IL-6). A confirmatory analysis comparing the differentially expressed genes identified in 2 separate analyses—MODS versus Control and NoMODS versus Control gave consistent results in both pathway and biofunction analyses (Fig 7A & 7B).

In immune cell type deconvolution, few markers were identified within the MODS differentially expressed gene set but NK and dendritic cell markers were up-regulated in MODS (S3 Fig). NK cell signalling pathways were also highly represented in canonical pathway analysis (Fig 7B). In contrast, there was a differential down-regulation in neutrophil deconvolution signature genes in those patients who went on to develop MODS and essentially no hyperacute signal for MODS in transcriptome markers for T- and B cell populations (S3 Fig). Immune modules associated with these MODS genes were mostly unannotated in modules M6, 7, 8, and 9 (S3 Fig), again highlighting that inflammatory pathways were not preponderant in the MODS signature (principally modules M1 through M5). However, again, there was a signal in module analysis for overexpression in Cytotoxic/NK cell modules (M3.6, M8.46) and underexpression in neutrophils (M.15). A transcriptomic signature for later development of MODS is present in the hyperacute window after injury, shows a strong signal for cell death pathways, and implicates NK cells and neutrophil populations in this differential response.

## Discussion

Our analysis presents a rare insight into the hyperacute window of the acute response to critical injury. This clinically deterministic time period shows a unique signature of transcriptome regulation that is not predictable from later study. The hyperacute transcriptomic response to critical injury involves only 4% of the genome. This then changes over 24 hours in both character and scale to a widespread response, which some have termed a “genomic storm” [10]. The development of MODS appears to be set within the hyperacute window and appears to be primarily a differential response of cell death and survival pathways rather than an excessive pro-inflammatory state.

A focused, hyperacute response implies that the acute response to injury can be defined and potentially measured and therapeutically modulated. Once the “storm” is established, it is almost impossible to affect and can only be allowed to run its course. An important finding of our study is that the character of both the leukocyte populations and their transcriptomic responses are very different between 2 hours and 24 hours postinjury, and this is not simply a progression of response in terms of magnitude and signal cascades. The acute response to critical injury cannot therefore be understood by examination of late time points and must include the immediate postinjury phase of care.

The hyperacute response is characterised by up-regulation of predominately innate inflammatory pathways and down-regulation of some classically adaptive pathways. Ingenuity pathway and biofunction analysis, immune cell deconvolution, and modular analysis were all consistent in identifying an up-regulation of damage recognition and downstream inflammatory signalling pathways associated with the fundamental concepts of activation of pattern recognition receptors leading to activation of sterile inflammation. NK cells emerged as potentially central to the immediate response to critical injury. Innate lymphoid responses to trauma have been poorly studied in the literature and refer to defective or impaired NK function as patients are investigated late in the clinical course [34–36]. The early down-regulation of adaptive responses and up-regulation of immunosuppressive pathways needs further study but may help to explain the development of a self-tolerant or immunosuppressive phenotype in critically injured trauma patients [37–38].

By 24 hours postinjury, over one fifth of the transcriptome was differentially expressed compared to the hyperacute profile. This widespread activation has been referred to as a “genomic storm,” although previous reports suggested this affected an even greater proportion of around 80% of the transcriptome [10]. The difference in our study may be due to several factors, including differences in contemporary trauma resuscitation (e.g., avoidance of crystalloids), our use of leuko-depleted red cell transfusions, or our analytic methodology. It is not clear what proportion of this genomic storm is due to cascading activation of inflammation or due to changes in leukocyte populations themselves. We have shown that there are marked changes in circulating leukocyte populations between admission and 24 hours. Some cells are extravasating into tissues and others are being recruited from the bone marrow, liver, spleen, and other stores, including populations not usually present in significant proportions [15, 39]. These cells will bring with them their own transcriptomes, will contribute to the widespread changes seen at 24 hours, and may be responsible for the change towards down-regulation seen at this later time point. The innate inflammatory pathways of the hyperacute response were already undergoing down-regulation, the innate lymphoid cell response had disappeared, and adaptive responses in particular were becoming more predominant. The biofunction, pathway and module analyses showed widespread and consistent patterns of dysfunction at 24 hours. This suggests that whatever the source of the transcriptome, the acute response to injury induces profound and widespread pathophysiologic changes in circulating leukocytes deserving of the term “genomic storm.” The “flap of butterfly wings” in the hyperacute response was almost completely distinct from the later transcriptomic profile of the genomic storm and represents an important area for translational research.

The hyperacute window contained a discrete transcriptomic signature for the development of MODS that is not detectable at 24 hours postinjury. Previous studies focusing on later postinjury time points have failed to identify a specific transcriptomic pattern for MODS and concluded that it must be related to the magnitude of response only [10]. Beyond the hyperacute phase, we also found limited differentiation between patients based on their subsequent development of MODS. A cascading or 2-hit response would show as a progressive increase in the magnitude of the response, in a pattern similar to Fig 1A. The fact that the MODS signature is present immediately implies that this is due either to the nature of the injuries themselves, or that there is a preexisting propensity for a differential response to the same physical insult [40]. Patients with MODS had higher lactates and base deficits on admission, and shock is a known strong predictor of the development of MODS [41]. It is possible that the genomic response to shock is a principal driver of the changes seen in the hyperacute window—physiology, which, for the most part, will be reversed in survivors by 24 hours. Larger scale analyses will be required to determine the drivers of the differential hyperacute transcriptomic response associated with the development of MODS.

MODS is usually understood as a dysregulated or excessive inflammatory response. Classical inflammatory pathways investigated in the pathogenesis of trauma multiple organ failure such as IL-6, IL-8, and IL-10 were not differentially expressed in patients who would later develop MODS. However, differential responses in the regulation and downstream signalling of these pathways, for example, the STAT3, ERK, and IGF-1 pathways for IL-6, featured strongly in the analyses. A differential regulatory or downstream response to IL-6 and other inflammatory pathways may be deterministic for the development of MODS in critically injured trauma patients.

However, pathway analysis of the hyperacute transcriptomic response showed a much higher enrichment of pathways associated with cell survival and death such as apoptosis and necrosis pathways. In immune module analysis, those modules that were overrepresented in MODS were generally poorly annotated and not widely shared among the inflammatory disorders used to construct the resource [29]. Together, these findings suggest that the principle driver of MODS may be a differential activation of the cell death responses to injury rather than a classically inflammatory-driven disease. Therapeutic agents known to up-regulate survival pathways do appear to ameliorate the organ injury in experimental models of severe trauma [42], and our finding is consistent with this but requires further study. Differential responses in NK cells again appeared implicated in the development of MODS, focusing attention on the role of innate lymphoid populations in the acute response to injury [15]. The relative down-regulation of neutrophil markers was a surprising finding given the traditional understanding of the role of neutrophils in the development of multiple organ dysfunction [43–44], although further work is required to confirm this. The hyperacute window provided a very different insight into the pathogenesis of MODS than could be garnered from later investigations.

There are several limitations to this study. Although we saw strong transcriptomic signatures, our sample size was relatively small. We attempted to reduce the heterogeneity in the sample populations by selecting or excluding specific injury types and enriched the response by choosing only a critically injured patient cohort to examine. Further study of larger cohorts will undoubtedly expand on the initial findings of this paper. We also took samples only in the hyperacute window and at 24 and 72 hours thereafter. We therefore do not know how long the hyperacute window is, nor when and how the signature changes to the more stable transcriptomic response seen after 24 hours. Previous studies have either sampled late or across the initial 24-hour period. These studies have suggested that there are later genomic signatures that may discriminate between different MODS phenotypes [45]. Further study of intermediate and later time points after injury is therefore required. Further flow cytometry analysis is also required to examine cell subpopulations and activation markers associated with the observed transcriptomic responses. Finally, this is an analysis of the circulating compartment only, and our findings cannot therefore reflect changes in tissue at the site of injury or in the tissues at sites of organ dysfunction.

In summary, the hyperacute time period after injury holds a specific signature of the response to critical injury that is an important window into our understanding of sterile inflammation and the development of a differential response that can lead to poor outcomes such as MODS. Contrary to expectations, the MODS signal was strongest in the hyperacute window and suggested a role for differential activation of cell death pathways, involving especially the NK cell population. As trauma is one of the few diseases for which the time of initial insult is known, this provides a unique window into the biology of human inflammation. The findings suggest multiple new directions for discovery research and hold opportunities for translation in diagnostics, clinical trial design, precision medicine, and novel therapeutic and management approaches. Understanding the human hyperacute response of the coagulation system changed an entire resuscitation paradigm within a decade. It is possible that understanding the hyperacute inflammatory response will do the same for our approach to organ dysfunction and protection.

## Supporting information

S1 Text Study protocol. (DOCX)

S1 Table STROBE checklist. (DOC)

S2 Table Immune module analysis. (XLSB)

S1 Fig Flow diagrams illustrating outcomes in study cohorts. (A) Microarray patient cohort. (B) Flow cytometry patient cohort.
(DOCX)

S2 Fig Representative gating strategy for flow cytometry studies. (A-C) Following exclusion of debris, lymphocytes were identified based on forward and side scatter properties, and this was followed by exclusion of doublets. (D-E) Natural killer (NK) cells were identified using CD56(+) and CD3(-) after exclusion of CD19(+) and CD14(+) cells. (F-G) T cells were identified using CD4(+) and CD8(+) after gating on CD3(+) cells. (H) B cells were identified using CD19(+) and CD3(-).
(TIFF)

S3 Fig Immune cell deconvolution and module analysis of Multiple Organ Dysfunction Syndrome (MODS) versus NoMODS transcriptome. (A) Immune deconvolution heatmap shows poor differentiation between MODS and NoMODS patients. (B) Fewer markers specific to immune cell activation, but there is up-regulation of NK cell markers and down-regulation of neutrophil markers. (C) Immune module analysis reveals few annotated modules, consistent with differential activation of noninflammatory pathways. There is a preponderance of modules in sections 6.X-9.X.
(TIF)

## Abbreviations

AIS: Abbreviated Injury Scale
FDR: false discovery rate
IRIS: immune response in silico
ISS: Injury Severity Score
MODS: Multiple Organ Dysfunction Syndrome
NK: Natural Killer
NoMODS: did not develop MODS
SOFA: sequential organ failure assessment
