# Correction: Signatures of inflammation and impending multiple organ dysfunction in the hyperacute phase of trauma: A prospective cohort study

**DOI:** 10.1371/journal.pmed.1002694

**Published:** 2018-10-31

**Authors:** Claudia P. Cabrera, Joanna Manson, Joanna M. Shepherd, Hew D. Torrance, David Watson, M. Paula Longhi, Mimoza Hoti, Minal B. Patel, Michael O’Dwyer, Sussan Nourshargh, Daniel J. Pennington, Michael R. Barnes, Karim Brohi

Table 1 presented numbers for the initial samples collected, however some of these samples presented low RNA yield on extraction and were discarded. Therefore, there are several places throughout the manuscript that required updated numbers to reflect the number of samples that were included for the transcriptomics analysis. **Table 1**should appear as it does below.

**Table 1:** Patient demographics.

		Microarray Cohort					Flow Cytometry Cohort	
Demographics	Control^a^	Critical^b^	p-value^1^	NoMODS	MODS	p-value^2^	HV	Critical^b^
Number	6	28	–	16	12	–	9	34
Age†	40 (29–46)	38 (24–54)	0.99	33 (23–41)	54(35–64)	0.05	34 (30–42)	51 (33.5–61)
Male (%)††	83	79	1.00	88	67	<0.01	60	71
Time from injury to blood draw (mins)†	67 (47–100)	95 (72–111)	0.14	80 (68–97)	108(100–115)	0.04	–	105 (90–117)
**Injury characteristics**								
ISS†	1 (0–2)	34 (27–38)	<0.01	29 (27–33)	38(28–38)	0.06	–	38 (29–45)
SBP on admission†	134 (121–152)	105(93–131)	0.09	112 (105–134)	78(unrec-93)	<0.01	–	112 (73–132)
Preintubation GCS†	15 (14–15)	14 (13–15)	0.55	15 (13–15)	14 (12–15)	0.12	–	8 (3–14)
BD (mmol/L)†	−1.1 (-1.7–−0.6)	4.3(9.7–2.3)	0.01	2.5 (2.0–3.8)	10.2(7.7–16.4)	<0.01	–	7.3 (4.3–16.9)
Shock Index (HR/SBP)†	0.7 (0.5–0.7)	1.2(0.7–1.6)	<0.01	0.8(0.7–1.1)	1.7(1.4–13.6)	0.01	–	0.9 (0.7–1.1)
Lactate (mmol/L)†	2.2 (1.6–2.3)	3.7(1.8–6.8)	0.13	2.0 (1.4–4.1)	7.0(4.2–12.9)	<0.01	–	3.2 (2.1–9.9)
AIS Head and Neck†	0 (0–0)	0 (0–0)	0.45	0 (0–0)	0 (0–1)	0.50	–	2 (0–4)
AIS Face†	0 (0–1)	0 (0–0)	0.34	0 (0–0)	0 (0–1)	0.14	–	0 (0–1)
AIS Thorax†	0 (0–0)	4 (3–5)	<0.01	4 (3–5)	5(4–5)	0.30	–	3 (3–4)
AIS Abdo/Pelvis†	0 (0–0)	3 (0–4)	<0.01	3 (2–4)	1(0–3)	0.23	–	3 (2–4)
AIS Extremity/Pelvis†	0 (0–0)	3 (2–3)	<0.01	3 (2–3)	3 (3–3)	0.45	–	3 (2–4)
**Outcomes**								
28-day mortality (%)††	0	14	<0.01	0	33	<0.01	–	29
Infections (%)††	0	62	<0.01	50	83	<0.01	–	48
Hospital stay (days)†	4 (3–6)	19(12–30)	0.03	19(12–26)	21(14–30)	0.01	–	13 (2–45)
ICU stay (days)†	0	6 (1–11)	0.03	2(0–5)	14(10–17)	<0.01	–	6 (2–21)

Median (interquartile range) reported unless otherwise specified.

p-value comparisons in the microarray cohort include ^1^Control versus Critical and ^2^MODS versus No MODS using ^†^Mann-Whitney U Test and ^††^Fisher’s exact Test.

^a^Minor injured trauma patient (ISS 0–4),

^b^critically injured trauma patient (ISS ≥ 25).

AIS, Abbreviated Injury Scale; BD, base deficit; GCS, Glasgow Coma Score; HR, heart rate; HV, healthy volunteer; Unrec, Unrecordable blood pressure; ICU, Intensive care unit; ISS, Injury Severity Score; MODS, Multiple Organ Dysfunction Syndrome; No MODS, did not develop MODS; SBP, systolic blood pressure

**S1 Fig** has also been corrected in the light of this error, including the numbers throughout the manuscript which come from that figure.

The first and second sentences in the “Methods and findings” section of the abstract should read: “We performed whole blood transcriptome and flow cytometry analyses on a total of 62 critically injured patients (Injury Severity Score [ISS] ≥ 25) in the hyperacute time period within 2 hours of injury. We compared transcriptome findings in 28 critically injured patients with those of 6 patients with minor injuries (ISS < 4).” The first sentence of the second paragraph in the same section should read: “In the transcriptome cohort, 12 critically injured patients later developed MODS. Compared with the 16 patients who did not develop MODS (NoMODS), maximal differential expression was seen within the hyperacute window. In MODS versus NoMODS, 363 genes were differentially expressed on admission, compared to only 33 at 24 hours postinjury.”

The first bullet point in the “What did the researchers do and find?” section of the Author summary should read: “We studied 29,385 immune cell genes within whole blood samples obtained from 28 critically injured patients at admission (within 2 hours of injury) and compared these to samples obtained at 24 and 72 hours following injury.”

The third sentence in the second paragraph of the subsection entitled “Patient selection” in the materials and methods section should read: “Of these, 36 patients had samples at all 3 time points and formed the critical population for this study. The critical population was dichotomised based on the presence (MODS: 19 patients) or absence (NoMODS: 17 patients) of MODS.”

The third sentence in the first paragraph of the subsection entitled “Microarray protocols” within the “Data analysis” section of the materials and methods section should read: “Of the original 36 critical samples, low RNA yields prevented several patients from further analysis: MODS = 7 and No MODS = 1.”

The first paragraph of the Results section should read: “The demographics and injury characteristics of the patient groups are shown in Table 1 and S1 Fig. Critically injured patients in the microarray cohort were severely injured with a median ISS of 34, compared to the control cohort with a median ISS of 1. No patient in this cohort received a blood transfusion or any surgical intervention prior to sampling. The initial blood sample was taken immediately on arrival in the emergency department, within 30 minutes and a median of 95 minutes after injury (within 2 hours of injury in all patients).”

The caption of Fig 1 should read: “Fig 1. The hyperacute transcriptomic response to critical injury. (A) There is a focused genomic response to injury in the hyperacute window after trauma. Columns show log p-values of differentially expressed genes between 28 Critical and 6 Control patients at 0 hours postinjury (hyperacute window), and between Critical patients at 24 and 0 hours and at 72 and 0 hours. In the hyperacute window, 4% of transcripts (1,239 of 29,385) were differentially expressed. This expanded to 21.4% (6,294 transcripts) at 24 hours and 21% (6,177 transcripts) at 72 hours. (B) Cluster analysis of differentially expressed genes in critically injured patients (versus controls). All hyperacute samples clustered separately, whereas there was no differentiation between samples at 24 or 72 hours. (C) Principal component analysis of differentially expressed genes in Critical and Control patients at 0 (hyperacute window), 24, and 72 hours postinjury. Critical patients were well separated from control patients, and there was marked separation of the hyperacute samples.”

The caption of Fig 2 should read: Fig 2. Leukocyte subpopulation changes in the hyperacute response to injury. (A) Immune cell deconvolution showing overall % differential regulation of immune cell–specific markers (selected from the immune response in silico [IRIS] resource [see methods]) between critical and control patients at 0 hours. There was predominant up-regulation of neutrophil, monocyte, and natural killer (NK) cell markers, a mixed-response in dendritic cells, and down-regulation of B and T cells. (B) Hierarchically clustered heatmap of immune cell–specific/enriched markers (selected from the IRIS resource) in the 27 critical patients at 0 and 24 hours postinjury. (C) Flow cytometry analyses were consistent with deconvolution. There was increase in numbers of neutrophils and lymphocytes (principally NK cells) in the hyperacute window. By 24 hours, total lymphocytes and NK cells were below normal (as compared to healthy volunteers), and median T-cell counts had also fallen significantly below healthy control counts. (Total leukocyte counts: healthy volunteer: 5.8 (4.4–7.1) x 10^9^/L; 0 hours: 19.5 (13.0–23.1) x 10^9^/L; 24 hours: 10.4 (7.8–14.1) x 10^9^/L; 72 hours: 8.2 (6.6–11.7) x 10^9^/L. *p < 0.05, **p < 0.01, ***p < 0.001, ****p < 0.0001).”

The caption of Fig 5 should read: “Fig 5. Patients who develop Multiple Organ Dysfunction Syndrome (MODS) have a specific differential gene expression only in the hyperacute window. (A) Differential expression heatmap with columns showing log p-values of all differentially expressed genes between 12 critical patients who later developed MODS versus 16 patients who did not develop MODS (NoMODS) at 0 hours (hyperacute time point), 24 hours, and 72 hours postinjury. At 0 hours, 363 transcripts were differentially expressed between MODS and NoMODS patients, and only 33 and 28 transcripts at 24 and 72 hours, respectively. (B) Cluster analysis of differentially expressed genes in MODS versus NoMODs patients in the hyperacute window. MODS patients clustered separately apart from 3 patients, 2 of which had a mild clinical phenotype that rapidly resolved. (C) Principal component analysis of differentially expressed genes in MODS versus NoMODS patients at 0 (hyperacute window), 24, and 72 hours postinjury. There was strong separation in the transcriptomic response to MODS in the hyperacute window.”

## Supporting information

S1 Fig Flow diagrams illustrating outcomes in study cohorts. (A) Microarray patient cohort. (B) Flow cytometry patient cohort.
(DOCX)
