# Supplementary material for: Signatures of inflammation and impending multiple organ dysfunction in the hyperacute phase of trauma: A prospective cohort study
Source: PLoS Med. 2017 Jul 17;14(7):e1002352. doi: 10.1371/journal.pmed.1002352 (PMC5513400; doi:10.1371/journal.pmed.1002352)
Supplement: S1 Text — (DOCX) [file pmed.1002352.s001.docx]

**S1 Text: Study Protocol**

**Study Objective**

To investigate the response of the whole blood transcriptome in critically injured patients in the hyperacute time frame (within 2 hours of injury).

**Main Questions**

1. Does the hyperacute window show a specific response to critical injury that is smaller and more specific than the “genomic storm” [1] previously described at later timepoints?
2. If present, how does an early hyperacute response change or evolve into the later widespread activation of the transcriptome?
3. Is the later development of Mutliple Organ Dysfunction Syndrome (MODS) associated with to a differential response in the hyperacute window?

**Patient Selection**

We will analyse a cohort of injured patients where we have samples available in the hyperacute window, at 24 and at 72 hours after injury.

*Inclusion criteria:*

Patients will be eligible for inclusion if the meet the following criteria:

- Blunt mechanism of injury;
- Injury Severity Score (ISS) greater than or equal to 25 (Critically injured cohort);
- ISS less than or equal to 4 (Control cohort).

*Exclusion criteria:*

To counter the influence of blood products and iatrogenic intervention on the immune response, and the influence of severe traumatic brain injury on outcomes, the following exclusion criteria will be applied:

- Receipt of blood products prior to admission blood draw;
- Receipt of greater than 1000mls crystalloid prior to admission blood draw;
- Presence of severe traumatic brain injury (defined as a head abbreviated injury scale score greater than 3).

**Outcomes**

The primary outcome measure is the presence or absence of MODS. MODS will be defined as a sequential organ failure assessment (SOFA) score of greater than or equal to 5 on two or more consecutive days, excluding the first 48 hours [2-3].

**Planned Analysis Steps**

The following comparisons will be analysed:

1. Critically injured patients versus Control patients at admission, 24 hours and 72 hours.
2. Crtitically injured patients at admission versus 24 hours and 72 hours.
3. Patients with MODS versus patients with No MODS at admission, 24 hours and 72 hours.

The first planned analysis will aim to examine differences in leukocyte gene expression. Statistical analysis will be conducted in R using Illumina Genome Studio V2011.1. Principal component analyses and Euclidean distance heatmaps will be used to assess patient stratification and gene expression patterns. Genes with a false discovery rate ≤ 0.05 will be considered differentially expressed.

The second analysis will use Ingenuity pathway analysis (Qiagen, inc) to identify the biological mechanisms enriched in differentially expressed genes. Genes from the data set that meet a p<0.05 threshold will be mapped to biological functions and/or pathways in the Ingenuity Pathways Knowledge Base. The most enriched pathways will be used to create co-expression networks to assess progression of gene changes and their clustering patterns.

The third analysis will use immune cell deconvolution to estimate the immune cell composition in our samples and examine differential responses in leukocyte populations within the hyperacute window. The analysis will be performed using the Immune Response *in Silico* (IRIS) repository [4], and data will be presented using heatmaps of differentially expressed immune cell markers.

**Subsequent Analysis**

After the initial planned analyses, we sought to corroborate the immune cell deconvolution findings by examining circulating leukocyte subpopulations using flow cytometry. This study required the recruitment of a new cohort of patients. We also sought to further explore the biological processes driving the hyperacute phase in the original patient cohort by using Chaussabel Modules [5].

**Additional Analyses requested by Reviewers**

Following reviewer comments, a further analysis cohort was added to the Ingenuity pathway analysis, comparing MODS versus No MODS at admission:

(MODS versus Control) versus (No MODS versus Control)

This cohort was included to confirm the findings in the MODS vs NoMODS analysis and determine whether there were conserved differences between MODS and No MODS in the hyperacute window.

**References**

1. Xiao W, Mindrinos MN, Seok J, Cuschieri J, Cuenca AG, Gao H, et al; Inflammation and Host Response to Injury Large Scale Collaborative Research Program. A genomic storm in critically injured humans. J Exp Med. 2011;208(13):2581-90.
2. Antonelli M, Moreno R, Vincent JL, Sprung CL, Mendoça A, Passariello M, et al. Application of SOFA score to trauma patients. Sequential Organ Failure Assessment. Intensive Care Med. 1999;25(4):389-94.
3. Ciesla DJ, Moore EE, Johnson JL, Sauaia A, Cothren CC, Moore JB, et al. Multiple organ dysfunction during resuscitation is not postinjury multiple organ failure. Arch Surg. 2004;139(6):590-4.
4. Abbas AR, Baldwin D, Ma Y, Ouyang W, Gurney A, Martin F, et al. Immune response in silico (IRIS): immune-specific genes identified from a compendium of microarray expression data. Genes Immun. 2005;6(4):319-31.
5. Chaussabel D, Quinn C, Shen J, Patel P, Glaser C, Baldwin N, et al. A modular analysis framework for blood genomics studies: application to systemic lupus erythematosus. Immunity. 2008;29(1):150-64.
