# Supplementary figures and images for: Signatures of inflammation and impending multiple organ dysfunction in the hyperacute phase of trauma: A prospective cohort study
Source: PLoS Med. 2017 Jul 17;14(7):e1002352. doi: 10.1371/journal.pmed.1002352 (PMC5513400; doi:10.1371/journal.pmed.1002352)

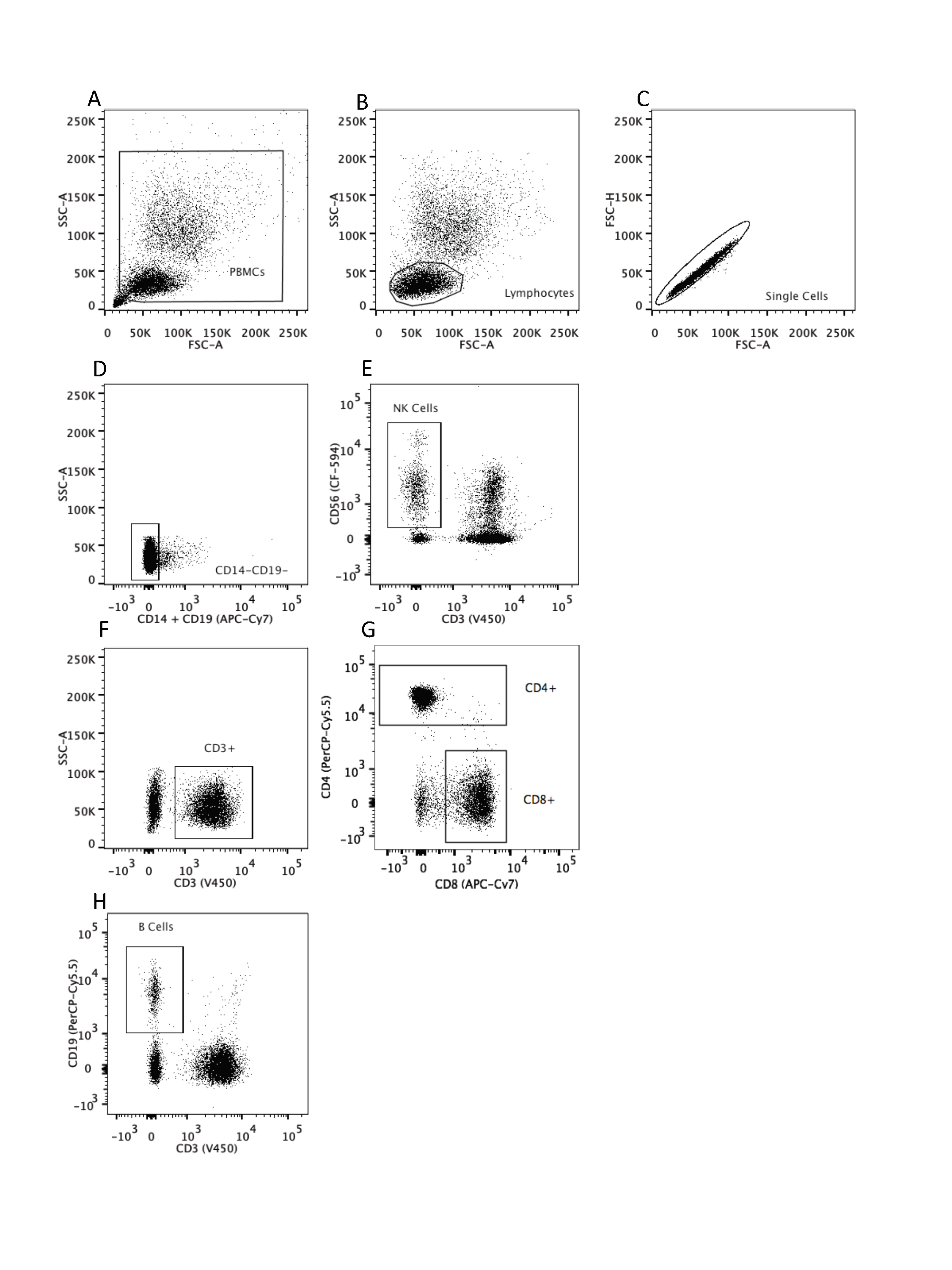

Supplement: S2 Fig — (A-C) Following exclusion of debris, lymphocytes were identified based on forward and side scatter properties, and this was followed by exclusion of doublets. (D-E) Natural killer (NK) cells were identified using CD56(+) and CD3(-) after exclusion of CD19(+) and CD14(+) cells. (F-G) T cells were identified using CD4(+) and CD8(+) after gating on CD3(+) cells. (H) B cells were identified using CD19(+) and CD3(-). (TIFF) [file pmed.1002352.s005.tiff]

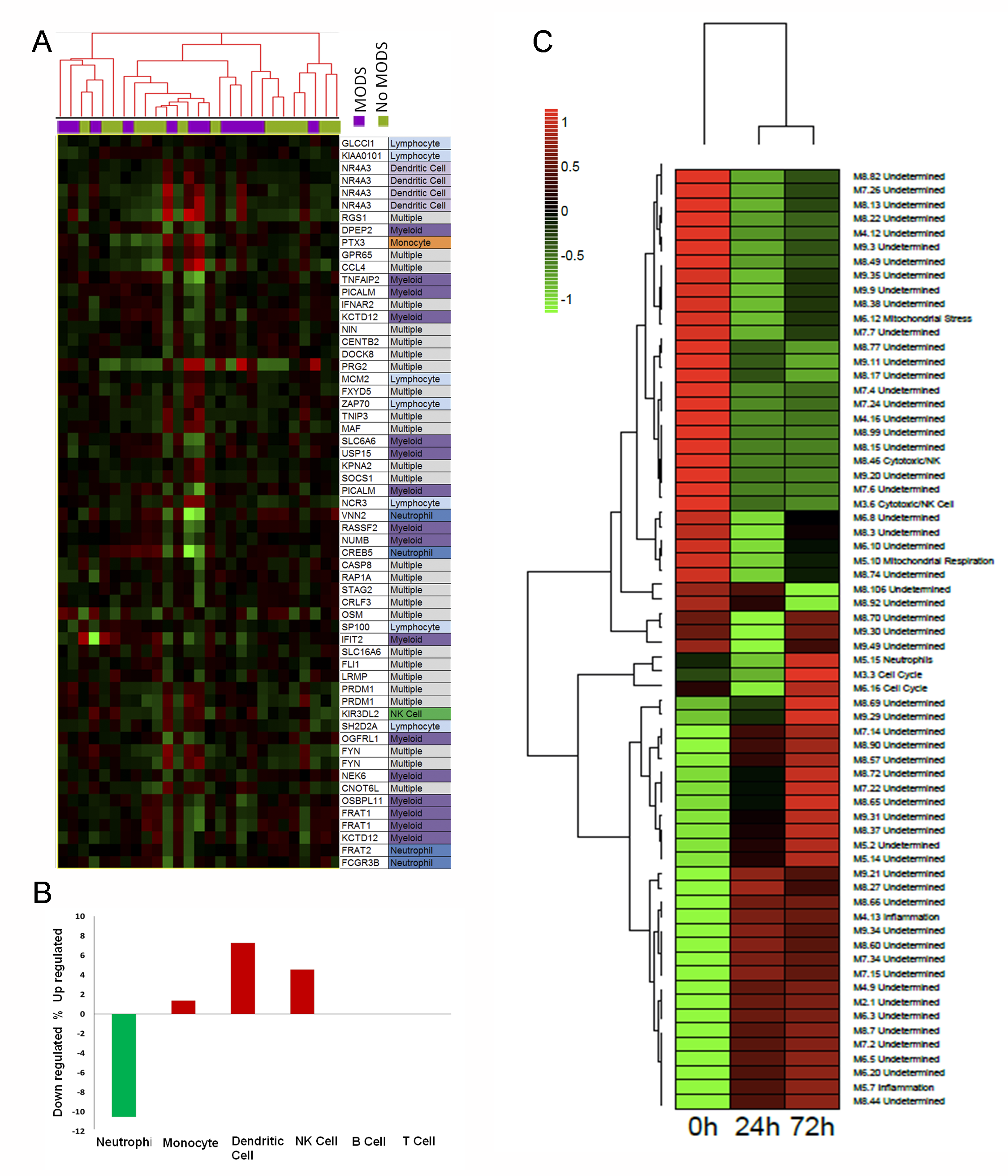

Supplement: S3 Fig — (A) Immune deconvolution heatmap shows poor differentiation between MODS and NoMODS patients. (B) Fewer markers specific to immune cell activation, but there is up-regulation of NK cell markers and down-regulation of neutrophil markers. (C) Immune module analysis reveals few annotated modules, consistent with differential activation of noninflammatory pathways. There is a preponderance of modules in sections 6.X-9.X. (TIF) [file pmed.1002352.s006.tif]
