# Supplementary material for: Signatures of inflammation and impending multiple organ dysfunction in the hyperacute phase of trauma: A prospective cohort study
Source: PLoS Med. 2017 Jul 17;14(7):e1002352. doi: 10.1371/journal.pmed.1002352 (PMC5513400; doi:10.1371/journal.pmed.1002352)
Supplement: Supplementary file 4 [file pmed.1002352.s004.docx]

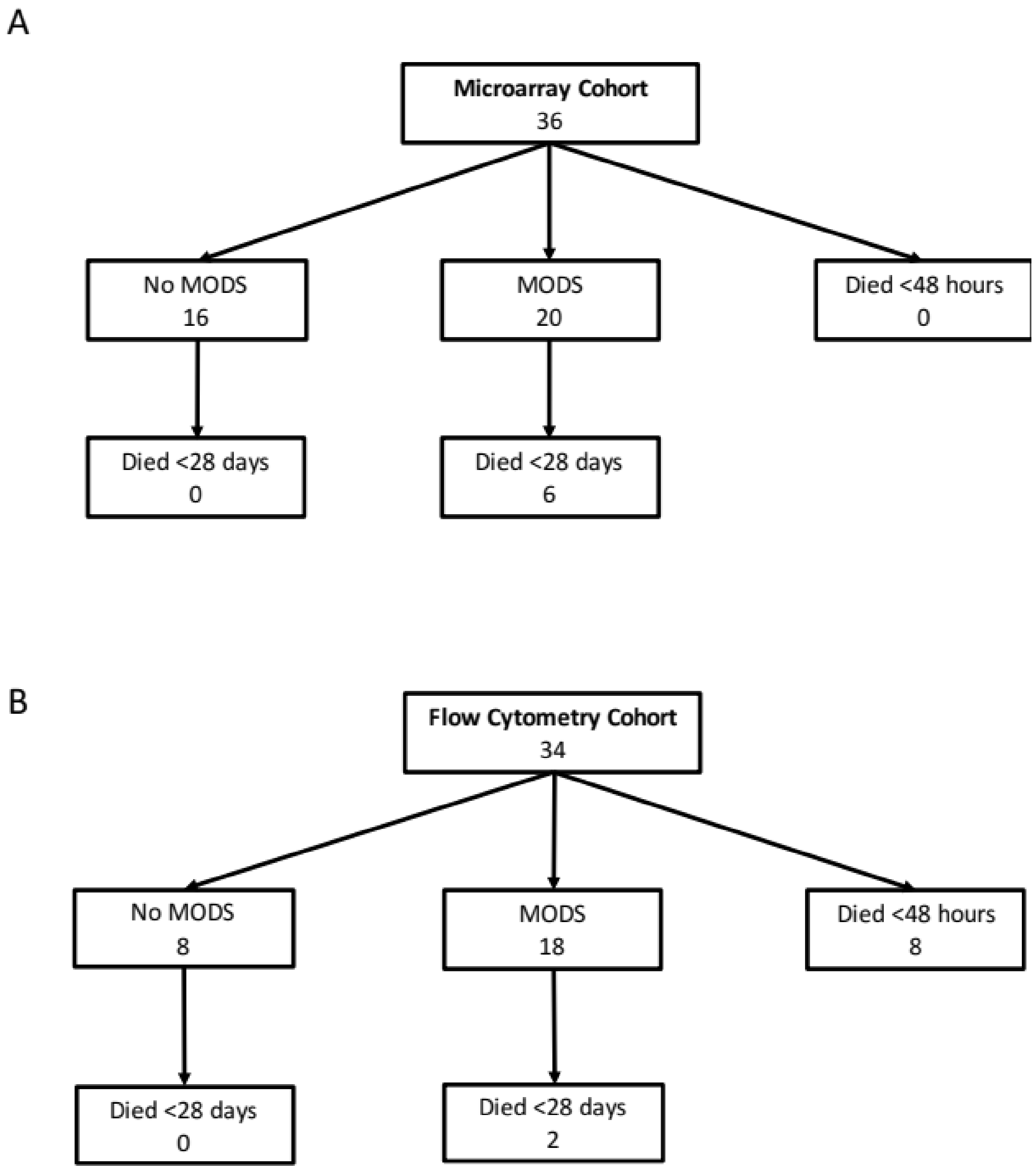
A
Microarray Cohort
36
No MODS
16
MODS
20
Died <48 hours
0
Died <28 days
0
Died <28 days
6
B
Flow Cytometry Cohort
34
No MODS
8
MODS
18
Died <48 hours
8
Died <28 days
0
Died <28 days
2
