# Supplementary material for: Correction: Signatures of inflammation and impending multiple organ dysfunction in the hyperacute phase of trauma: A prospective cohort study
Source: PLoS Med. 2018 Oct 31;15(10):e1002694. doi: 10.1371/journal.pmed.1002694 (PMC6209129; doi:10.1371/journal.pmed.1002694)

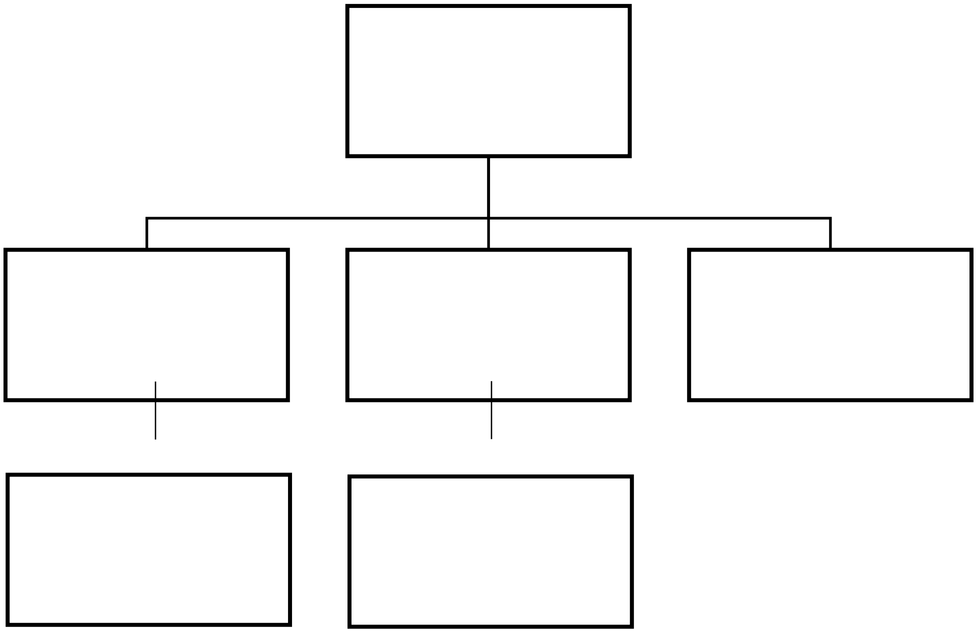

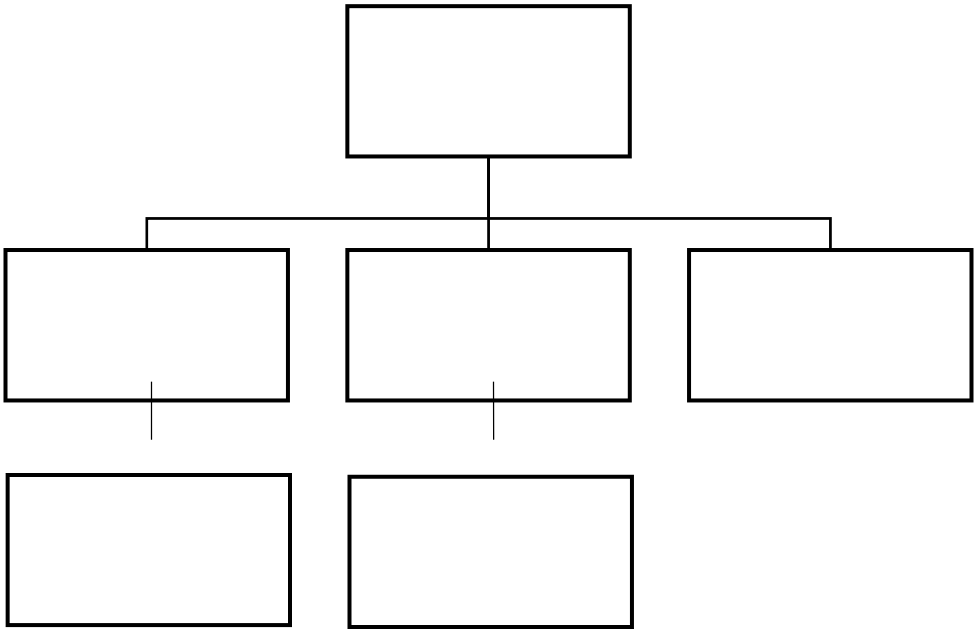

Supplement: Supplementary file 1 [file pmed.1002694.s001.docx]
